# Impact of mucin on the anti-erosive/anti-abrasive efficacy of chitosan and/or F/Sn in enamel in vitro

**DOI:** 10.1038/s41598-021-84791-9

**Published:** 2021-03-05

**Authors:** Benedikt Luka, Vivien Arbter, Kathrin Sander, Andrea Duerrschnabel, Nadine Schlueter

**Affiliations:** Division for Cariology, Department of Operative Dentistry and Periodontology, Center for Dental Medicine, Medical Center - University of Freiburg, Faculty of Medicine, University of Freiburg, Hugstetter Str. 55, 79106 Freiburg, Germany

**Keywords:** Preventive dentistry, Biopolymers, Tooth wear

## Abstract

The application of stannous ions in combination with fluoride (F/Sn) is one of the central strategies in reducing erosive tooth wear. F/Sn efficacy can be enhanced by adding chitosan, a positively charged biopolymer. For patients with low saliva flow, this efficacy, however, is not sufficient, making further improvement desirable. This could be achieved by combining chitosan with other molecules like mucin, which together might form multilayers. This in-vitro study aimed to investigate the effect of chitosan, mucin, F/Sn and combinations thereof on enamel erosion and erosion-abrasion. Human enamel samples (n = 448, 28 groups) were cyclically eroded or eroded-abraded (10 days; 6 × 2 min erosion and 2 × 15 s/200 g abrasion per day). Samples were treated 2 × 2 min/day with solutions containing either, chitosan (50 or 500 mPas), porcine gastric mucin, F/Sn or combinations thereof after abrasive challenge. Tissue loss was measured profilometrically, interaction between hard tissue and active agents was assessed with energy dispersive spectroscopy and scanning electron microscopy. Chitosan and F/Sn showed the expected effect in reducing tissue loss under erosive and under erosive-abrasive conditions. Neither mucin alone nor the combinations with mucin showed any additional beneficial effect.

## Introduction

Erosive tooth wear is caused by the combined impact of chemical (acids) and physical factors, (e.g. toothbrushing) on the dental hard tissue^1^. Over the last decades, its prevalence increased and it is a common disease in the general population with a world prevalence amongst adolescents and adults by approximately 20% to 45%^2^. Fluoride ions in combination with stannous ions (F/Sn) have proven to be potent agents in reducing erosive^3,4^ and erosive-abrasive dental hard tissue loss^5^. Its effectiveness is based on the incorporation of stannous ions into the superficial parts of both enamel and dentine as well as on the formation of acid resistant, tin-rich precipitates on the tooth surfaces. The more tin is retained in and on the dental hard tissue, the less demineralisation will occur^6^. Further improvement in efficacy of stannous and fluoride ion containing formulations was achieved by adding the biopolymer chitosan to F/Sn preparations, both to solutions used under in vitro conditions^7^ and to toothpastes used under in vitro^5^ and in situ conditions^8^. This improvement is mainly attributed to a more pronounced retention of tin on the tooth surface^7^, although other synergistic effects of chitosan and F/Sn^9^ as well as interactions between the polymer and the dental hard tissue may also play a role. Chitosan is a positively charged, linear chained polysaccharide. The polymer binds to surfaces with a negative zeta potential^10^. Therefore it is likely that chitosan layers also develop on the negatively charged enamel surfaces, which can function as pH stable barriers against acids^11^; furthermore, it is discussed that these layers may have lubricating effects reducing friction between abrasives, toothbrush and dental hard tissue, thus decreasing abrasive tissue loss.

Whilst under oral conditions with normal salivary flow the effect of chitosan, stannous and fluoride ion containing preparations is undisputed and outstanding, there are indications that the active agents are considerably less effective in case of xerostomia^12^, maybe due to lack of specific polymers from the saliva. As patients with xerostomia are at high risk not only for carious lesions but also for demineralisation processes of the dental hard tissue in general, there is a clear need to improve effectiveness of common demineralisation inhibiting preparations for patients with low saliva flow. Mucins, another group of biopolymers seem to be promising for this purpose since they play an important role in the lubricating properties of saliva, which is lacking in case of dry mouth^13^. In particular the mucosa cell protecting effect of porcine gastric mucin in the acidic environment in the stomach might be of interest. Under nearly neutral conditions (pH 6), porcine gastric mucin is in a liquid state allowing a simple application of the active agent. Under acidic conditions (pH 2) the solution becomes viscous, maybe leading to more protective properties if retained on the surface^14^ but is probably more difficult to apply. Mucins are made up of a core protein with glycosylated oligosaccharides^15^. Whilst non-glycosylated parts of mucins have a neutral charge^16^, highly glycosylated parts are negatively charged^15^.

If oppositely charged polymers, such as chitosan and mucin, are applied alternatingly, multilayers of these polymers could be formed^17^ potentially enhancing the anti-erosive effects of chitosan by reinforcing the barrier against acids. Formation of multi-layers of the positively charged chitosan and negatively charged bovine submaxillary mucin has already been shown on hydrophilic silica^18^.

The aim of the present in vitro study was to further enhance the anti-erosive/anti-abrasive efficacy of F/Sn with combinations of biopolymers which could potentially prevent severe demineralisation. Therefore the effect of two chitosans of different molecular weights and one mucin from porcine gastric mucosa on their own and in combinations on tissue loss in human enamel either under erosive or erosive-abrasive conditions was investigated. In two separate experiments the effects of the biopolymers on their own or in combination with F/Sn were evaluated.

The first research hypothesis was that the application of chitosan or mucin or combinations thereof show different effects in reduction of erosive and erosive-abrasive tissue loss in enamel in vitro in the absence of F/Sn. The second research hypothesis was that a mucin-additive influences the anti-erosive/anti-abrasive efficacy of the active agents chitosan or F/Sn as well as the combination of both.

## Results

### Dropouts

Ten of the 448 samples (see Table 2 for group assignment) could not be analysed. From experiment 1 without F/Sn, under erosive conditions, one sample in the negative control (no application of any experimental solution containing F/Sn or biopolymers), two samples in the positive control (F/Sn only), two samples in the group treated one after another with chitosan 50 and with mucin (Ch50Mu) and one sample in the group treated one after another with chitosan 500 and with mucin (Ch500Mu) were not analysable. From experiment 2 with F/Sn, under erosive conditions, one sample in the positive control (F/Sn only), two samples in the group treated with mucin and F/Sn only (Mu) and from experiment 2, under erosive-abrasive conditions, one sample in the group treated with chitosan 50 and F/Sn only (Ch50) were not measurable. Failures were due to partial or complete loss of coverages on the reference area, damaging of the experimental surface, incorrect baseline measurement or other measuring difficulties.

### Tissue loss measurement

**Figure 1:**
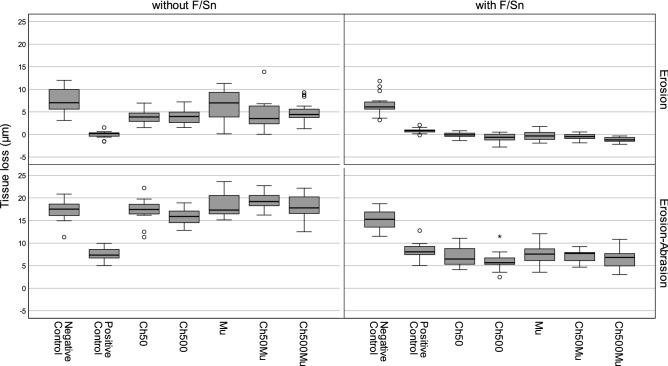
Box-whisker-plots of the profilometric tissue loss (µm). The left diagrams show results from experiment 1 in which F/Sn was only used in the positive control, the right diagrams show results from experiment 2 in which F/Sn was applied in all groups except for the negative control. The upper diagrams show groups only treated with citric acid solution (erosion), the lower diagrams show groups which were additionally abraded (erosion-abrasion). Negative Control = without active agents; Positive Control = F/Sn only; Ch50/Ch500 = chitosan with viscosities of 50 mPas/500 mPas; Mu = porcine gastric mucin; Ch50Mu/Ch500Mu = combination of chitosan and mucin in separate solutions (application order chitosan first, mucin second). The circles represent outliers that lie within the 1.5- to 3-fold interquartile distance, asterisks represent extreme values that lie outside of the threefold interquartile distance.

Results of tissue loss measurement are shown in Table 1 and in the box-whisker-plots in Fig. 1 (see Supplementary Data S1 for set of data). Under erosive conditions in experiment 1 (without F/Sn), only Ch50 (p = 0.005) and Ch500 (p = 0.008) showed an effect in reduction of tissue loss compared to the negative control, which was not treated with active agents; however, both chitosans reached with a reduction by 47% and 46%, respectively, not the efficacy level of the positive control (F/Sn only), which completely prevented tissue loss (p ≤ 0.001). Mucin did not reduce tissue loss, neither if used in combination with Chitosan (Ch50Mu: p = 0.173; Ch500Mu: p = 0.145) nor on its own (p = 1.000), compared to negative control. Under erosive-abrasive conditions in experiment 1 without F/Sn no statistically significant reduction of tissue loss was reached by any of the experimental solutions (p values ranging between 0.151 and 1.000).

**Table 1:** Results of profilometry (mean tissue loss ± SD in µm) and EDX analysis (amount of carbon and tin in weight percent (mean wt% ± SD)).

	Tissue loss (µm)		Carbon (wt%)		Tin (wt%)	
	Erosion	Erosion-abrasion	Erosion	Erosion-abrasion	Erosion	Erosion-abrasion
**Experiment 1 without F/Sn**						
Negative control	^A^7.7 ± 2.8^a^	^B^17.3 ± 2.4^a,c^	^A^8.9 ± 1.1^a^	^A^7.7 ± 1.5^a,c^	^A^0.2 ± 0.1^a^	^B^0.1 ± 0.1^a^
Positive control	^A^0.0 ± 0.8^b^	^B^7.6 ± 1.4^b^	^A^13.9 ± 1.6^b^	^B^10.4 ± 1.2^b^	^A^17.2 ± 2.0^b^	^B^3.6 ± 0.7^b^
Ch50	^A^4.0 ± 1.5^c^	^B^17.2 ± 2.6^a,c^	^A^10.7 ± 1.0^c^	^B^7.8 ± 0.7^a,c^	^A^0.1 ± 0.1^a^	^A^0.0 ± 0.0^a^
Ch500	^A^4.1 ± 1.8^c^	^B^15.9 ± 1.5^a^	^A^10.6 ± 1.1^a,c^	^B^7.0 ± 0.5^a^	^A^0.1 ± 0.2^a^	^A^0.1 ± 0.1^a^
Mu	^A^6.8 ± 3.3^a,c^	^B^18.3 ± 2.6^a,c^	^A^9.4 ± 1.2^a,c^	^B^7.3 ± 0.8^a,c^	^A^0.1 ± 0.1^a^	^A^0.0 ± 0.1^a^
Ch50Mu	^A^4.4 ± 3.3^a,c^	^B^19.4 ± 1.7^c^	^A^16.0 ± 1.8^b^	^B^8.2 ± 0.5^c^	^A^0.1 ± 0.1^a^	^A^0.1 ± 0.2^a^
Ch500Mu	^A^4.9 ± 2.3^a,c^	^B^18.0 ± 2.6^a,c^	^A^13.8 ± 2.2^b^	^B^8.6 ± 1.4^a,b,c^	^A^0.1 ± 0.1^a^	^A^0.2 ± 0.2^a^
**Experiment 2 with F/Sn**						
Negative control	^A^6.6 ± 2.4^a^	^B^15.2 ± 2.1^a^	^A^12.4 ± 2.1^a^	^B^9.1 ± 1.8^a,b^	^A^0.1 ± 0.2^a^	^A^0.1 ± 0.1^a^
Positive control	^A^0.8 ± 0.5^b^	^B^8.3 ± 1.7^b^	^A^17.6 ± 1.0^b^	^B^8.2 ± 0.8^a,b^	^A^14.4 ± 0.7^b^	^B^3.7 ± 0.3^b,d^
Ch50	^A^-0.1 ± 0.5^c^	^B^6.9 ± 2.0^b,c^	^A^17.6 ± 1.7^b^	^B^8.8 ± 0.4^a^	^A^14.6 ± 1.0^b^	^B^4.5 ± 0.5^c^
Ch500	^A^-0.7 ± 0.9^c,d^	^B^6.0 ± 2.0^c^	^A^18.2 ± 1.0^b^	^B^8.4 ± 0.5^a,b^	^A^13.8 ± 0.9^b^	^B^3.8 ± 0.4^b,c^
Mu	^A^-0.3 ± 1.0^c,d^	^B^7.5 ± 2.1^b,c^	^A^18.3 ± 1.7^b^	^B^8.0 ± 0.3^b^	^A^10.2 ± 0.8^c^	^B^3.2 ± 0.3^d,e^
Ch50Mu	^A^-0.5 ± 0.6^c^	^B^7.1 ± 1.4^b,c^	^A^17.8 ± 1.2^b^	^B^8.2 ± 0.3^a,b^	^A^10.7 ± 0.8^c^	^B^3.1 ± 0.4^e^
Ch500Mu	^A^-1.2 ± 0.6^d^	^B^6.6 ± 2.0^b,c^	^A^17.4 ± 1.4^b^	^B^8.5 ± 0.8^a,b^	^A^10.6 ± 0.9^c^	^B^3.3 ± 0.6^b,d,e^

Experiment 1 = no F/Sn except for positive control; experiment 2 = application of F/Sn in all experimental groups. Negative Control = without active agents; Positive Control = F/Sn only; Ch50/Ch500 = chitosan with viscosities of 50 mPas/500 mPas; Mu = porcine gastric mucin; Ch50Mu/Ch500Mu = combination of chitosan and mucin in separate solutions. Different lowercase letters indicate statistically significant differences between groups within one experiment (experiment 1 or experiment 2) and one condition (erosion or erosion-abrasion). Significant differences between corresponding groups of the two conditions (erosion and erosion-abrasion) are marked with differing capital letters.

In experiment 2 the addition of F/Sn to all groups completely prevented tissue loss in all groups under erosive conditions without abrasion (p ≤ 0.001). Precipitates were found in most cases. Under erosive-abrasive conditions with F/Sn all experimental biopolymer solutions reduced tissue loss significantly by between 51 and 61% compared to negative control (p ≤ 0.001) and were, except for Ch500, comparable to the positive control (0.232 ≤ p ≤ 0.997). The addition of Ch500 increased the efficacy of F/Sn only (positive control) significantly by 16% (p = 0.021).

Additional abrasion increased tissue loss significantly in all groups in both experiments, whether without or with F/Sn compared to erosive conditions (p ≤ 0.001).

### Energy dispersive x-ray spectroscopy (EDX)

Results of the EDX analyses are shown in Table 1 (see Supplementary Data S2 for set of data).

Differences in carbon content most likely correspond to different amounts of organic material deposition on the surfaces, which in the present study can stem from either the biopolymers or amine fluoride used in the F/Sn solution.

In experiment 1 without F/Sn under erosive conditions carbon content was, except for the Mu and the Ch500 group, significantly higher after biopolymer or F/Sn application than in the negative control (no treatment with active agents) with an increase of 20 to 80% in these groups (p ≤ 0.05). Abrasive impact decreased carbon content distinctly and only in the positive control (F/Sn only) significantly more carbon (+ 35%) than in the negative control was found (p ≤ 0.001). When adding F/Sn to all groups in experiment 2 the carbon content in general was higher and was significantly increased by application of all solutions independent of the presence of biopolymers. The amount of carbon was reduced by abrasive impact with no difference between groups, except for Ch50 and Mu.

The tin measured by EDX on the sample surfaces stems from the stannous chloride in the F/Sn solution. As F/Sn was used in all groups in experiment 2 tin was detected on all surfaces, with comparable values in the F/Sn only (positive control) and F/Sn/chitosan groups under erosive conditions. The content was significantly lower in all mucin treated groups compared to F/Sn only by 26–29% and compared to F/Sn/chitosan by 23–30% (p ≤ 0.001). After abrasive impact, only the Ch50 group showed higher Sn values than the group applying F/Sn only (positive control). Again the additional use of mucin reduced the tin retention compared to the respective chitosan on its own, with a statistically significant difference between Ch50 and MuCh50. However, in general the differences in these groups were small.

**Figure 2:**
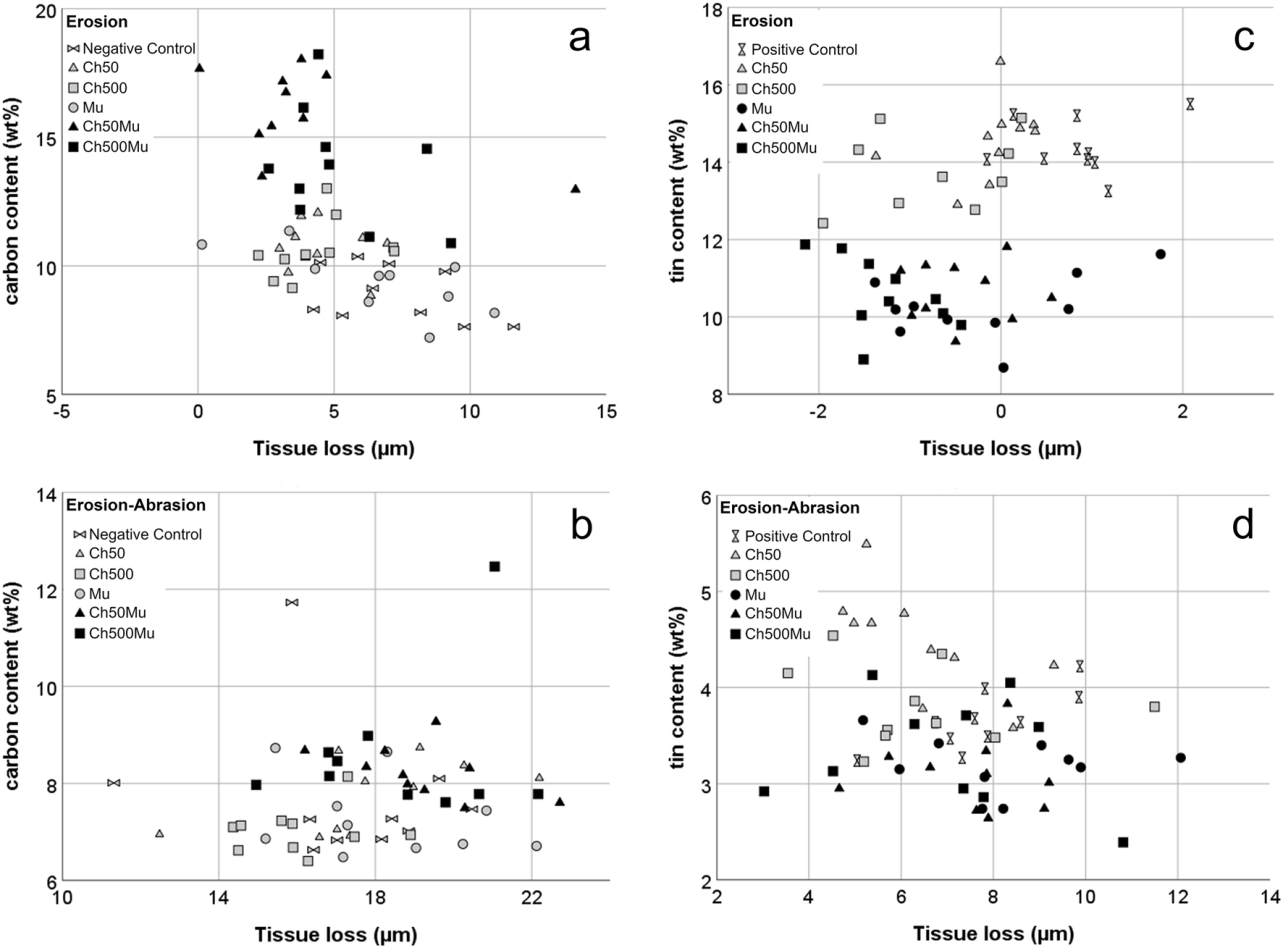
Scatterplot of results of tissue loss in relation to carbon content (left column (**a**/**b**), groups of experiment 1 only; no stannous ions) and to tin content (right column (**c**/**d**), groups of experiment 2; treated with F/Sn) under erosive (upper row; **a**/**c**) and erosive-abrasive conditions (lower row; **b**/**d**). For carbon content no positive control (F/Sn only) is displayed; for tin content no negative control (without active agents) is displayed. The dots are marked according to group assignment. Negative Control = without active agents; Positive Control = F/Sn only; Ch50/Ch500 = chitosan with viscosities of 50 mPas/500 mPas; Mu = porcine gastric mucin; Ch50Mu/Ch500Mu = combination of chitosan and mucin in separate solutions (application order chitosan first, mucin second). For the carbon content the results are split into one polymer (grey) and two polymers (black). For tin content the results are marked according to the charge of the last applied agent (grey: positively charged; black: negatively charged). After application of two polymers the amount of carbon increased; the retained amount of tin decreased in case of negative charge of the last applied agent.

Arranging the groups of experiment 1 (without F/Sn) to three strata where no, one and two biopolymers were used, the additional analysis showed a significant impact of the number of polymers on the amount of carbon (Fig. 2). While under erosive conditions there was a significant difference in carbon content between the application of no, one and two biopolymers (p = 0.005), under erosive-abrasive conditions there was only a difference for the application of one and two polymers (p ≤ 0.001), but not for the comparison between one and no polymer as well as between two and no polymer (n.s.). Splitting the groups of experiment 2 (with F/Sn) into two strata according to the charge of the last applied active agent (Fig. 2), the analysis revealed that the tin amount was higher if the charge of the last applied agent was positive (p ≤ 0.001), both under erosive and erosive-abrasive conditions. This means that the application of mucin either on its own or in combination resulted in less amounts of tin on the surfaces.

### Scanning electron microscopy (SEM)

**Figure 3:**
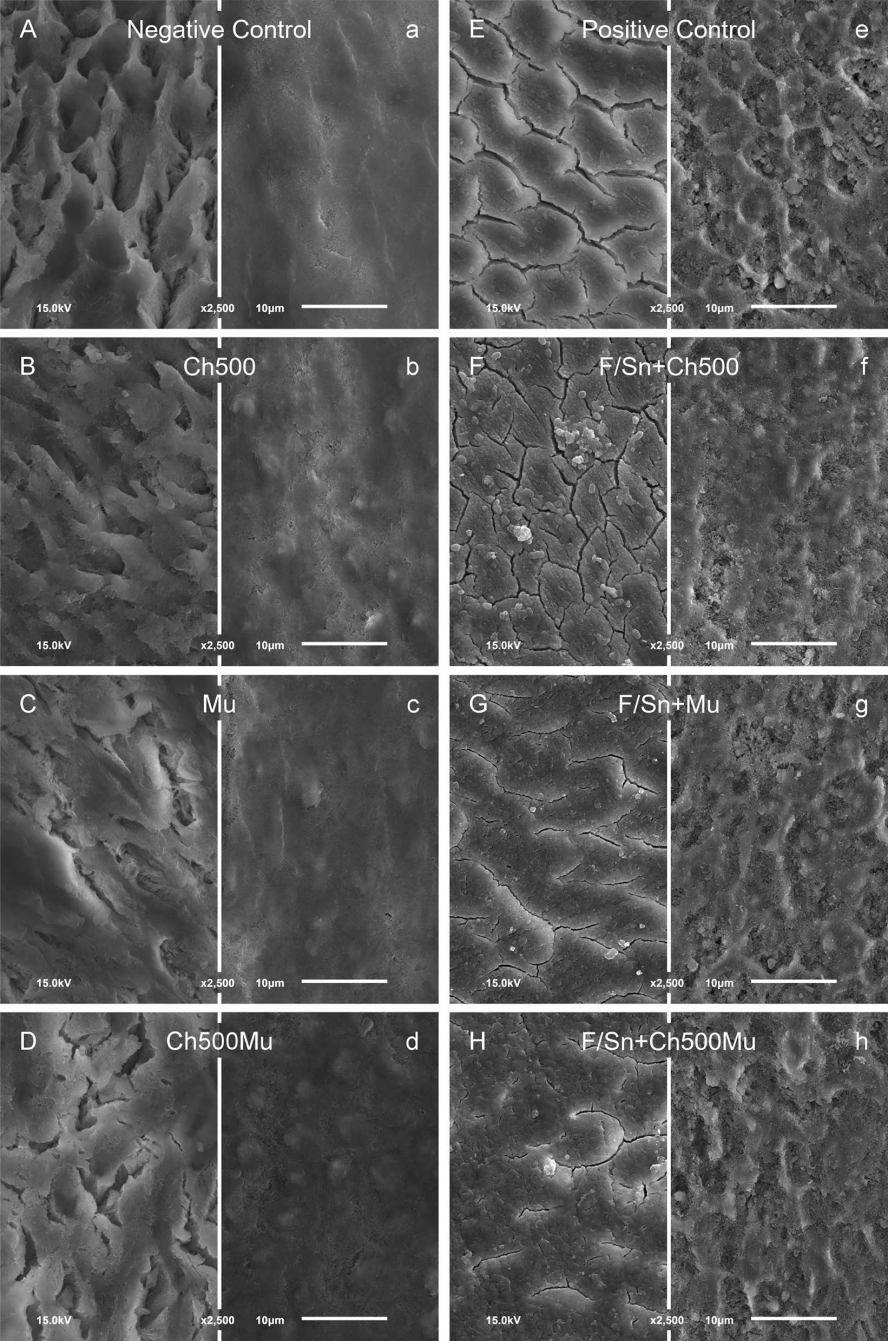
SEM images of selected samples. The left column (**A**–**D**/**a**–**d**) depicts samples from experiment 1 without application of F/Sn, the right column (**E**–**H**/**e**–**h**) depicts samples from experiment 2 with application of F/Sn. Samples under erosive conditions are marked with capital letters, samples under erosive-abrasive conditions are marked with lowercase letters. Negative Control = without active agents; Positive Control = F/Sn only; Ch500 = chitosan with viscosity 500 mPas; Mu = porcine gastric mucin; Ch500Mu = treated with Ch500 and Mu; F/Sn = additional application of F/Sn (original magnification: 2,500-fold).

Representative SEM pictures of negative control (without active agents), positive control (F/Sn only), Ch500, Mu and Ch500Mu are shown in Fig. 3. As there was no difference between the Ch50 or Ch50Mu and Ch500 or Ch500Mu groups, the results of the Ch50 and Ch50Mu groups are not shown. In general, the surfaces of groups after erosion are more structured; the surfaces after erosion-abrasion show a more flattened appearance. The eroded only negative control shows the typical appearance of etched enamel. Without the use of F/Sn (experiment 1) after erosion there are some amorphous precipitates visible on the surfaces in the mucin treated groups. The surface of the chitosan group shows a surface comparable to those which were eroded only (negative control). After application of the stannous ion containing solutions without abrasion the typical formation of a continuous surface precipitate can be seen in all groups, independent of the biopolymer applied.

In groups where additional abrasion was applied, all surfaces treated with polymers but not with stannous ions show an amorphous appearance. On all surfaces treated with stannous ions a flattened etching pattern is visible, slightly covered with an amorphous precipitate in the Ch500 and Ch500Mu group.

## Discussion

The study investigated for the first time the direct impact of mucin on the anti-erosive and anti-abrasive effect of F/Sn and chitosan containing solutions. The erosion-abrasion-model was intended to resemble erosive tooth wear caused by an erosive diet, e.g. frequent consumption of soft drinks. The application order of erosive and active agents should mimic the use of mouthrinses after the daily brushing procedure. The protocol followed current recommendations for erosion-abrasion-models^19,20^ and the exact setting was chosen in accordance with previous studies^7^ to obtain comparability. The F/Sn-concentration was chosen according to the F/Sn-concentration in a commercially available mouth rinse with an anti-erosive claim representing the gold standard in erosion therapy^21^.

The chitosan solutions with medium sized molecules (150 kDa/50 mPas and 350 kDa/500 mPas) were most effective in previous investigations and were therefore used in the present study^7,22^. Porcine gastric mucin is easily available and has similar properties to the high molecular mucin MUC5B found in saliva^23^. The concentration of 0.27% has been used according to previous erosion studies^24,25^. In groups combining positively and negatively charged active agents the positively charged chitosan and/or F/Sn were always applied first, in order to enable an initial adherence to the negatively charged enamel surface. The positively charged chitosan or stannous ion containing solution and the negatively charged mucin solution were applied separately, since chitosan or stannous ions and mucin can react with each other if mixed together, forming mucin-chitosan or mucin-stannous ion complexes^26^. Chitosan and stannous ions can be applied together; it is well known that they do not hamper each other in efficacy or bioavailability.

We decided against including native saliva to obtain information about reactions between the single components used and their retention behaviour on the dental hard tissue without being masked by any other macromolecule. Follow-up studies including saliva, preferably under in situ conditions, in which the efficacy under normal and low saliva flow conditions is compared, have to be planned in case of promising results.

Tissue loss of both the negative control (without active agents) and the positive control (F/Sn only) was in the range found in the literature^5,7,27,28^, making the results comparable with previous studies and showing the robustness of the study model. Even if there are some differences between the control groups of both experiments, these differences regarding tissue loss are quite small and within the normal variability. The reason for higher differences in carbon content between control groups is not clear at this moment. Therefore, the interpretation of the results was performed without directly comparing the results between both separately performed experiments 1 (without F/Sn) and 2 (with F/Sn).

A striking finding of this study is that chitosan without F/Sn and without any further active ingredients added, reduced erosive tissue loss by nearly 50%. This is remarkable considering the low native pH of the chitosan solutions. Therefore, this study has clearly shown that even acidic chitosan solutions do not increase erosive tissue loss in enamel at all. In fact the low pH might have even led to a stronger adhesion of chitosan. In solutions with a low pH chitosan molecules have more protonated amine groups, resulting in a stronger positive zeta-potential^29^. These positively charged chitosan molecules can directly bind to negatively charged surfaces^10^. Indeed EDX results show higher amounts of carbon compared to the negative control (significant at least for Ch50; p = 0.058 for Ch500). The only possible sources for carbon were amine fluoride in the F/Sn solution, biopolymer molecules and the enamel itself. As the carbon content of eroded enamel was observed without active agents in the negative control and in experiment 1 amine fluoride was only applied in the positive control, increased levels of carbon most likely originated from the biopolymers. Therefore EDX results support the suggestion that chitosan formed layers on the negatively charged enamel surface. Once established, these layers could probably have acted as barriers against the impacting acids^11^.

Unlike chitosan, mucin was not able to reduce the erosive tissue loss. This result is in contrast to a study^30^, showing a protective effect of mucin used as an additive to an artificial saliva solution in a single application and an erosion model inducing very initial erosion. But it is in accordance with a study comparing the effects of mucin and casein on the reduction of enamel surface microhardness by erosion^25^. Mucin can bind to both hydrophobic and hydrophilic surfaces^31^ with its neutral, non-glycosylated parts^32^. But the affinity of mucin to hydrophilic surfaces like enamel is lower than to hydrophobic surfaces due to electrostatic repulsion by the negatively charged glycosylated parts of mucin^33^. In the present study the pH of the mucin solution was adjusted to 7 to obtain a solution with no erosive potential. At pH 7, however, mucin is folded, hiding its non-glycosylated parts by shielding them with its charged glycosylated parts towards the aqueous solution, as opposed to mucin at pH 2, where it is less charged and unfolded^34^. It was intended to unfold its tertiary structure during the next erosive challenge in order to increase reactivity. Independent of the molecule’s conformation mucin was apparently not able to sufficiently adsorb to the negatively charged enamel. This is confirmed by the EDX measurement, showing no higher carbon content in the mucin group compared to the group not treated with any active agent (negative control).

Decher et al. introduced the idea of forming multilayers by alternating application of oppositely charged polyelectrolytes in 1992^17^. The present study is the first attempt to apply this approach on dental hard tissue. The positively charged chitosan and the negatively charged mucin have already shown their potential for interaction in solutions^26^ as well as their ability to build multilayers on negatively charged silica and mica^18,32^. The EDX results of the present study indicate that deposition of biopolymer complexes might also take place on enamel. The amount of carbon measured by EDX was higher for the groups combining chitosan and mucin compared to the groups in which only one polymer was used. It is conceivable that there was some kind of interaction between the polymers, leading to higher amounts of carbon on the surface. This also corresponds to the altered surface morphology. The capacity to decrease erosive tissue loss, however, seems not to depend on the sheer mass of carbon on the surface.

An additional rationale behind applying polymers was to reduce friction effects of the toothbrush and the toothpaste. Mucin is known for its lubricating effects^35^. In some studies, chitosan also showed a certain degree of lubrication^36^ perhaps being able to prevent abrasion. However, under erosive-abrasive conditions without F/Sn (experiment 1) all preparations completely lost their efficacies. The lubricating effects of chitosan seem to play a role, if chitosan is added to toothpastes, where abrasive particles can be coated with chitosan layers, reducing the abrasive effect of these particles^37^. However, when chitosan was used to coat the dental hard tissue, the lubricating effect, if any, was very limited. Maybe the order of application in the erosion-abrasion cycle plays a role. The polymers were applied after abrasion and should reduce erosion-abrasion in the following cycles. Presumably the binding forces of the chitosan^38^ and mucin^31^ to the enamel surface were too weak to withstand the abrasive forces. It would be worth investigating whether the application of the biopolymers before the abrasive challenge can increase efficacy.

As expected F/Sn on its own showed a good anti-erosive efficacy in experiment 2. It is known that stannous ions not only precipitate on the surface but are also incorporated into the erosively softened superficial enamel. The amount of tin incorporated in and retained on the surface of enamel is the key mechanism of its anti-erosive potential.

By the additional use of one of the biopolymers or biopolymer-combinations, erosion was even completely inhibited in the present study. An improvement by chitosan has already been shown in previous studies, which was attributed to an increase in tin retention on the surface, induced by chitosan^5^. However, in the present study the amount of tin retained on the enamel surface was smaller as expected with only Ch50 under erosive-abrasive conditions leading to a statistically significant increase. In all groups treated with mucin the amount of retained tin even decreased significantly. However, despite the reduction of retained tin the efficacy of F/Sn was not decreased. Probably the anti-erosive effect of the biopolymers already shown without F/Sn (experiment 1) was added to the effects of F/Sn compensating the reduction of tin retention. It could also be speculated that chitosan can be stabilized by the complexation with polyvalent metal ions^39^. Therefore probably not the amount but the binding forces of stannous ions and biopolymers were increased. However, the processes taking place on the enamel surface, when biopolymers and F/Sn are applied need further research.

Conclusively, the findings of this study confirm the well-known benefits of chitosan as an additive to F/Sn both under erosive and erosive-abrasive conditions. Furthermore, an anti-erosive but not anti-erosive/anti-abrasive efficacy could be shown for chitosan itself, without F/Sn; mucin, on the contrary, had no anti-erosive or anti-erosive/anti-abrasive effect. Therefore, the first research hypothesis has been confirmed only in parts. The effect of F/Sn, of chitosan and of combinations thereof was not enhanced by the addition of mucin. Therefore the second research hypothesis has to be rejected. The aim to find combinations of polymers which are more effective than chitosan alone failed. In particular the reduced retention of tin on the surfaces is undesirable, as the retained amount of tin is a key point in efficacy of F/Sn containing preparations, especially if thinking about long term effects. Therefore, adding the most abundant polymer from saliva—muci—to F/Sn and chitosan containing preparations seems not to be an advantageous way for developing more effective demineralisation inhibiting preparations.

## Materials and methods

**Figure 4:**
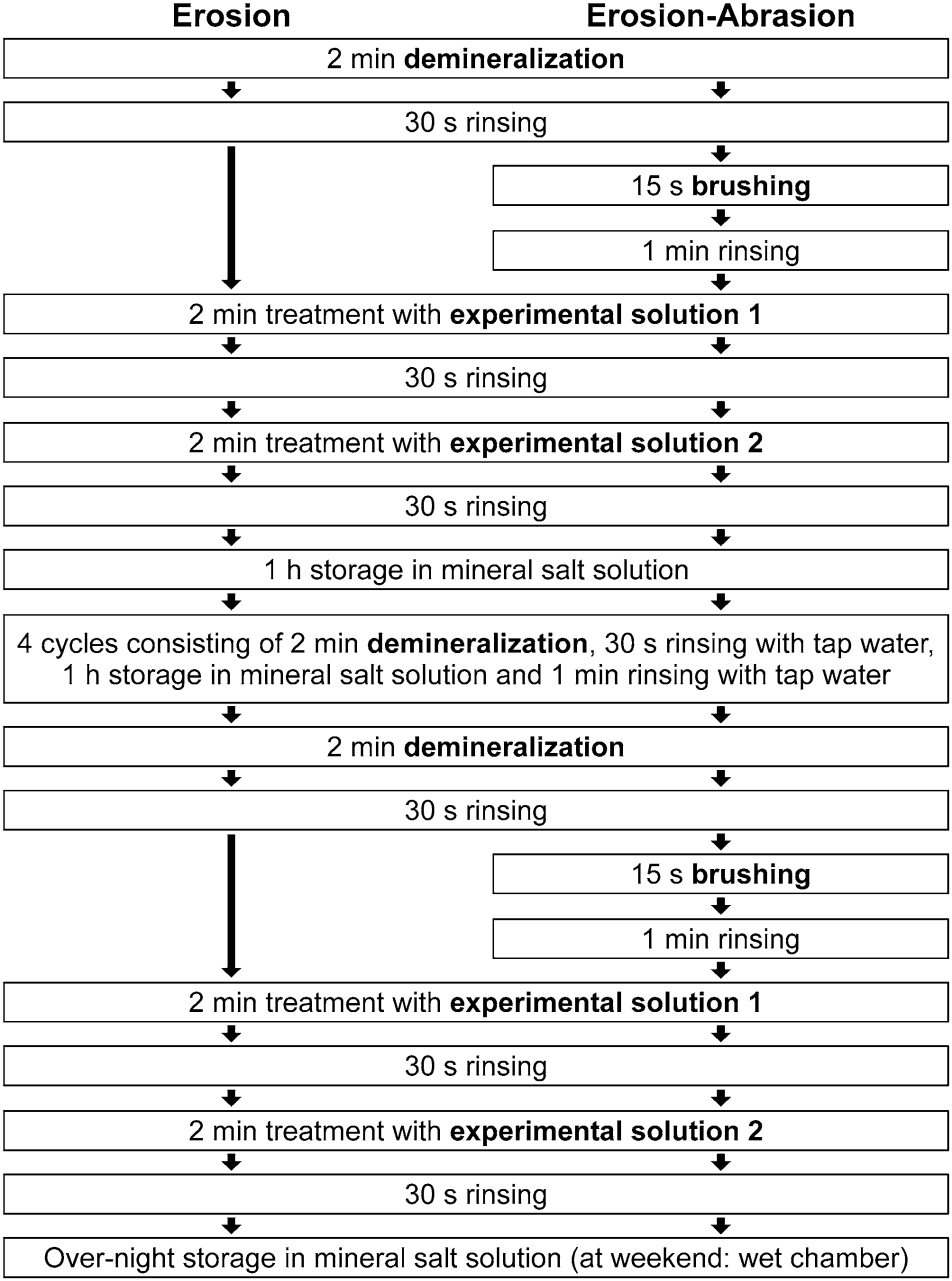
Flow chart of study procedures in each Experiment. Except for negative and positive control group, in experiment 1 samples were treated with biopolymers only, in experiment 2 samples were additionally treated with F/Sn. The two different biopolymers were always applied in two separate solutions; in experiment 2 the F/Sn was applied together with chitosan. See Table 2 for experimental solutions used in the respective group and experiment.

In 2 experiments, with 2 conditions (erosion and erosion-abrasion) and 7 different applications of polymers a total of 28 groups (2 × 2 × 7) of human enamel samples were exposed to cyclic erosion or erosion-abrasion. Experiment 1 was conducted without impact of stannous ions in combination with fluoride ions (F/Sn), in experiment 2 F/Sn was applied additionally. In both experiment 1 and experiment 2 half of groups were treated with solutions only (erosive conditions), the other half were brushed before application of experimental solutions (erosive-abrasive conditions). Polymers under investigation were two different types of chitosan (Ch50: viscosity 50 mPas and Ch500: viscosity 500 mPas) and mucin (Mu; porcine gastric mucin). In each experiment seven different forms of application were used: singular application of biopolymers (Ch50, Ch500, Mu) either without F/Sn (experiment 1) or in combination with F/Sn (experiment 2) or combined application of biopolymers in the order chitosan first, mucin second (Ch50Mu, Ch500Mu) either without F/Sn (experiment 1) or in combination with F/Sn (experiment 2). In both experiments F/Sn without any additive (positive control) or no active agent (negative control) served as control groups (Fig. 4 and Table 2).

**Table 2:** Experimental solutions used in the respective group and experiment.

Group	Negative control	Positive control	Ch50	Ch500	Mu	Ch50Mu	Ch500Mu
**Experiment 1 (without F/Sn)**							
Experimental solution 1	–	F/Sn	Ch50	Ch500	Mu	Ch50	Ch500
Experimental solution 2	–	–	–	–	–	Mu	Mu
**Experiment 2 (with F/Sn)**							
Experimental solution 1	–	F/Sn	Ch50 + F/Sn	Ch500 + F/Sn	F/Sn	Ch50 + F/Sn	Ch500 + F/Sn
Experimental solution 2	–	–	–	–	Mu	Mu	Mu

*F/Sn* 500 ppm F^−^ and 800 ppm Sn^2+^; pH 4.4. *Ch50* 0.5% chitosan (Viscosity: 50 mPas); pH 4.6. *Ch500* 0.5% chitosan (Viscosity: 500 mPas); pH 4.6. *Mu* 0.27% mucin (mucin from porcine stomach Type II); pH 7.0. *Ch50 + F/Sn* 0.5% chitosan (Viscosity: 50 mPas), 500 ppm F^−^ and 800 ppm Sn^2+^; pH 4.6. *Ch500 + F/Sn* 0.5% chitosan (Viscosity: 500 mPas), 500 ppm F^−^ and 800 ppm Sn^2+^; pH 4.6

### Sample preparation

Approximately 150 previously impacted human third molars, which had no contact to oral cavity and oral hygiene products prior to the extractions, were used to prepare a total of 448 enamel samples as already described elsewhere^27^, which were randomly assigned to 28 groups (n = 16 per group). Sample size calculation was performed with G*Power 3.1.9.4 on the basis of previous studies^7,40^ with α = 0.05 and 1 − β = 0.8. If a 10% reduction is classified as a relevant effect size, 14 samples would be necessary in each group. With a safety margin a sample size of 16 was used.

The teeth used were all extracted for medical reasons in different dental offices; informed consent was obtained from all donors. All teeth were immediately pooled and anonymously given in saturated thymol solution, whereby an assignment to a person was no longer possible. All methods were carried out in accordance with relevant guidelines and regulations. The collection and use of human teeth in the study followed the Declaration of Helsinki (2013) and were approved by the local ethics committee (Ethik-Kommission Albert-Ludwigs-Universität Freiburg, No. 352/16). The study was planned and performed according to the Guidelines for Safeguarding—Good Research Practice—of the German Research Foundation.

Longitudinal enamel slices were cut. The slices were ground flat and polished with silicon carbide abrasive paper P1′200 and P4′000 removing approximately 300 µm of the outer enamel. Samples with a surface area of approximately 2 mm × 3 mm were cut and checked for flatness; a deviation of 1.5 µm was accepted. All samples were checked for demineralisation, fluorosis and cracks under a light microscope with 16-fold magnification. Samples were mounted on sample holders for a brushing machine with light curing resin. Baseline measurements were conducted. Afterwards one half of each sample surface was covered with light curing resin to serve as reference area. Between the processing steps and until use all samples were stored in a wet chamber at 4 °C.

### Solutions and preparations

For demineralization a 0.5% citric acid solution was used (native pH 2.4, 5 g/L citric acid monohydrate dissolved in distilled water).

In-between procedures and overnight, samples were stored in a mineral salt solution according to Gerrard and Winter (pH 6.8)^41^, containing mineral in concentrations comparable to saliva (4.08 mmol H_3_PO_4_ (Ortho-Phosphoric acid 99%), 11.90 mmol NaHCO_3_ (sodium hydrogen carbonate), 20.10 mmol KCl (potassium chloride) and 1.98 mmol CaCl_2_ (calcium chloride)) but no organic additives such as proteins or polymers.

For the F/Sn solution 4 ml amine fluoride (RonaCare Olaflur, Merck, Darmstadt, Germany) were mixed with 100 ml distilled water to which 306 mg SnCl_2_ (tin (II) chloride dehydrate, Merck, Darmstadt, Germany) were added. If the solution was used on its own, the final concentrations of 500 ppm F^-^ and 800 ppm Sn^2+^ (pH 4.4) were reached by adding 100 ml of distilled water^7^. When used in combination with chitosan, the solution was diluted with the respective chitosan solution instead of distilled water.

The chitosan-solutions were prepared by dissolving 1 g of the respective Chitosan (Ch50 (viscosity: 50 mPas; molecular weight: 150 kDa) or Ch500 (viscosity: 500 mPas; molecular weight: 350 kDa); Chitoscience, Chitosan of crustaceans, degree of deacetylation 80%; Heppe Medical Chitosan GmbH, Halle (Saale), Germany) in 100 ml of 0.5% acetic acid. If chitosan was used on its own (experiment 1, without F/Sn), 100 ml of distilled water were added, resulting in a concentration of 0.5% chitosan (pH 4.6). If chitosan was used in combination with F/Sn (experiment 2) 100 ml of the F/Sn-solution were added resulting in 0.5% chitosan, 500 ppm F^-^ and 800 ppm Sn^2+^ (pH 4.6)^7^.

The mucin-solution was prepared by dissolving 2.7 g of mucin (from porcine stomach Type II, crude mixture of glycoproteins; sugar and amino acid composition of the mucin has not been determined by the manufacturer; M2378, Sigma Aldrich, Steinheim, Germany) in 1000 ml distilled water (0.27%). pH was adjusted to 7.0 by adding NaOH^25^.

Abrasion was performed with fluoride free toothpaste slurry (Lavera Neutral Zahngel, RDA 91.5 ± 2.1 (Value determined in the School of Dentistry, Oral Health Research Institute, Indiana University)^7^, Lavera GmbH & Co.KG, Wennigsen, Germany). The toothpaste was mixed with distilled water 1:3 by weight (pH 7.1).

### Procedures

Samples were assigned to 28 groups from which 14 groups were allocated to experiment 1 (without F/Sn) and 14 groups to experiment 2 (with F/Sn). In each experiment half of groups (7 in experiment 1 and 7 in experiment 2) were treated with acids (erosive conditions) and the other half (7 in experiment 1 and 7 in experiment 2) was treated with acids and was additionally brushed (erosive-abrasive conditions).

All samples were demineralized with the citric acid solution 6 times per day for 2 min over a total of 10 experimental days (2 × 5 days) as described elsewhere^7^. After each demineralization samples were rinsed for 30 s with tap water. The samples under erosive-abrasive conditions were brushed every day twice, after the first and the last demineralization, for 15 s in an automated brushing machine (linear movement pattern, 200 g load, 25 oscillations, 13 mm travel path, 60 mm/s travel velocity) with a manual toothbrush (elmex 39 toothbrush medium, CP GABA GmbH, Hamburg, Germany) and toothpaste slurry. Afterwards, the samples were rinsed with tap water for 60 s in order to remove all slurry remnants. The other half of groups (erosive conditions) was not brushed. Subsequently, also twice daily, all samples, except for the negative control, were immersed in the experimental solutions for 2 min. According to group assignment either one polymer (Ch50, Ch500 or Mu) or one of the chitosans consecutively followed by mucin (Ch50Mu, Ch500Mu) was applied. Chitosan and mucin were always used as separate solutions in order to avoid any reaction between both polymers. In experiment 2 F/Sn (500 ppm F^-^ and 800 ppm Sn^2+^) was additionally applied in each group, either as additive to the chitosan solution^7^ or as a separate solution in groups without chitosan. The positively charged active agents (F/Sn, Ch50, Ch500) were always applied first and the negatively charged (Mu) second. In both experiments the F/Sn solution without polymers was used as a positive control. After each immersion in an experimental solution, samples were rinsed with tap water for 30 s. After treatment with experimental solutions all samples of all groups including negative control were stored in the mineral salt solution for 1 h in-between cycles and over-night and rinsed for 60 s before further treatment. All treatments with solutions were performed in a shaking water bath at 25 °C with gentle agitation (35 horizontal movements per min). At weekend and after the last experimental day until analysis samples were stored in a wet chamber at 4 °C (Fig. 4 and Table 2).

### Tissue loss measurement, scanning electron microscopy, energy dispersive x-ray spectroscopy

The coverages of the reference areas were carefully removed with a scalpel without touching the samples’ surfaces. All samples were controlled regarding scratches and damage. The tissue loss measurement was conducted with an optical profilometer and the measuring software FRT Acquire as described elsewhere^7,27^. The same settings were used for the baseline measurement and measurement after the last experimental day (chromatic white light sensor; 1000 Hz; 200 pixels per 3 mm). Same area for analysis was found via an external reference point located on each sample holder. At each measurement time point, three parallel traces of 3 mm length were measured at intervals of 0.2 mm and analysed. On each trace two parallel regression lines with 0.5 mm of length were constructed, one on the reference area and one on the experimental area, both at least 0.3 mm off the margin between reference and experimental area. The vertical distance between the two regression lines was measured. The baseline value of each trace was subtracted from the value obtained after the last experimental day on the respective trace. Tissue loss per sample was defined as mean of the three values per sample.

For reproducibility ten repeated measurements were conducted of the same two samples repositioning them for each measurement. At a mean tissue loss of 19.6 µm for one sample the standard deviation was 0.1 µm. For the other sample at a mean tissue loss of 1.0 µm the standard deviation was also 0.1 µm.

Ten samples of each of the 28 groups were randomly selected for energy dispersive x-ray spectroscopy (EDX), from which three were selected after EDX measurement for scanning electron microscopy (SEM). The samples were mounted on holders for SEM, dried at ambient air and sputtered with gold (sputter time: 60 s; amperage: 60 mA). EDX and SEM analyses were both performed with the JSM-IT100 LA (Jeol Germany Freising) and the corresponding software JSM-IT100 (Vers. 1.09; Jeol Germany Freising). A secondary electron detector was used for structural analysis. For all analyses acceleration voltage of 15 kV and a working distance of 10 mm was used. All other settings including take-off angle, tilt angle, probe current, counts per second and scanning mode were kept constant.

For EDX the standardless analysis was used. The relative amount of elements on the surface (mass or weight percent; wt%) was quantified at 1,000-fold magnification. The elements of interest were carbon, oxygen, phosphate, calcium and tin. The values for gold were measured but not quantified. Only results of carbon and tin are presented in Table 1. The surface morphology was analysed by SEM at 2,500-fold magnification.

### Statistical analysis

All quantitative data were checked for normal distribution (Shapiro–Wilk-test). As no significant deviation was found, One Way ANOVA with Tamhane’s post hoc (significant deviation from the homogeneity of variances, Levene’s test) was performed for comparison of groups within one experiment (with F/Sn or without F/Sn) and one condition (erosion or erosion-abrasion). The comparisons of the corresponding groups of the conditions erosion and erosion-abrasion within one experiment were performed with t-tests for independent samples and Bonferroni correction (level of significance p ≤ 0.007).

For the experiments without F/Sn (experiment 1) additionally the amount of carbon on the surfaces was analysed according to the number of polymers applied. For this purpose the groups per condition (erosion or erosion-abrasion) were subsumed (no polymer: negative control; one polymer: Ch50, Ch500 and Mu; two polymers: Ch50Mu, Ch500Mu). As the positive control contained two relevant elements (tin and carbon) this group was excluded from this analysis. For the experiments with F/Sn (experiment 2) additionally the amount of tin on the surfaces was compared according to the charge of the last active agent applied. For this purpose the groups per condition (erosion or erosion-abrasion) were subsumed (positive: F/Sn, Ch50, Ch500 or negative: Mu, Ch50Mu, Ch500Mu). As the negative control contains no tin, this group was excluded from the analysis. Comparison of subsumed groups was performed with independent sample t-test and Bonferroni correction (level of significance p ≤ 0.0125).

Level of significance was set to 0.05 if not otherwise stated.

### Statement of ethics

All donors gave their informed consent for using the teeth. All teeth were used anonymously. This procedure and the study protocol were approved by the local ethics committee (Ethik-Kommission Albert-Ludwigs-Universität Freiburg, No. 352/16).

## Supplementary Information

Supplementary Information 1.

Supplementary Information 2.

## Data Availability

All data generated or analysed during this study are included in this published article and its Supplementary Information files (see Supplementary Data S1 and S2).
